# Advantageous effects of rumen-protected phytonutrients from tropical plant extracts on rumen fermentation efficiency and methane mitigation using *in vitro* fermentation technique

**DOI:** 10.5713/ab.24.0576

**Published:** 2024-10-28

**Authors:** Maharach Matra, Chaichana Suriyapha, Gamonmas Dagaew, Rittikeard Prachumchai, Srisan Phupaboon, Sukruthai Sommai, Metha Wanapat

**Affiliations:** 1 Division of Animal Science, Department of Agricultural Technology, Faculty of Technology, Mahasarakham University, Maha Sarakham 44150, Thailand; 2 Tropical Feed Resources Research and Development Center, Department of Animal Science, Faculty of Agriculture, Khon Kaen University, Khon Kaen 40002, Thailand; 3 Department of Animal Science, Faculty of Agricultural Technology, Rajamangala University of Technology Thanyaburi, Pathum Thani 12130, Thailand

**Keywords:** Feed Additive, Feed Innovation, Fruits, Natural Compounds, Ruminants

## Abstract

**Objective:**

Tropical plants are composed of phytonutrients (PTNs) and are utilized for their capacity to manipulate rumen fermentation characteristics and methane production. The aim of this experiment was to determine the impact of microencapsulated PTNs-extracted from lemongrass and mangosteen peel (M-LEMANGOS), as well as crude protein (CP) levels on nutrient degradability, rumen ecology, microbial population, and methane emission in an *in vitro* study.

**Methods:**

The treatments were randomly assigned in a 2×4 Factorial arrangement in a completely randomized design. The two factors consisted of CP percentage in the concentrate diet (16% and 18% CP) and the levels of M-LEMANGOS addition (0%, 2%, 4%, and 6% of the total substrate).

**Results:**

The results showed that nutrient degradability both 12 and 24 h were significantly increased with M-LEMANGOS at 4% total substate. In part of volatile fatty acids (VFAs), particularly propionate and total VFA, these were enhanced by %CP and M-LEMANGOS combination. The %CP increased ruminal ammonia-nitrogen concentration (NH_3_-N), while M-LEMANGOS supplementation reduced such concentration. Methane production and *Methanobacteriales* population at 12 and 24 h were reduced when supplemented with M-LEMANGOS at 4% total substate. The population of *Fibrobacter succinogenes, Ruminococcus flavefaciens*, and *Megasphaera elsdenii* were increased with the interaction between %CP and M-LEMANGOS addition.

**Conclusion:**

M-LEMANGOS indicates promising potential as a plant-based PTN for dietary modulation of rumen fermentation and mitigation of methane production.

## INTRODUCTION

The phenomenon known as the greenhouse effect has garnered heightened attention in recent years due to its role in the increasing global temperatures. The three main greenhouse gases are carbon dioxide (CO_2_), methane (CH_4_), and nitrous oxide (N_2_O), respectively. In comparison to CO_2_, CH_4_ has a 28-fold higher global warming potential over a century. This expansion has detrimental effects on animals, humans, and the environment [1]. Animal production accounts for 14.5% of greenhouse gas emissions caused by human activity in the agricultural sector. It is responsible for around 37% of global CH_4_ emissions, mostly caused by ruminal enteric fermentation in ruminants [2]. Hence, reducing CH_4_ emissions from ruminants will have a greater impact on managing greenhouse gas levels in the livestock production system. Currently, the presence of plant-based phytonutrients (PTNs) has been demonstrated to inhibit the process of methanogenesis in the rumen. Nevertheless, significant discrepancies exist among research investigating the impact of PTNs on rumen fermentation and methane production [3].

Tropical climate fosters the growth of a large number of evergreen fruit trees. The consistent nature of this environment promotes the production of tropical fruits that are able to meet the demands of the world market. The annual output of tropical fruits reflects their global availability and demand [4]. The worldwide popularity and demand for the annual production reflects tropical fruits. Agricultural fruit wastes such weed plants, peels, and seeds are produced in significant amounts as fruit agriculture develops and these wastes turn into a challenging environmental problem [5]. Peels, kernels, and seeds from fruits wastes typically contain a significant quantity of PTNs and it could be utilized as a source of beneficial antioxidant substances [5]. The most consumed tropical fruits are pineapple, papaya, jackfruit, mango, as well as mangosteen. Mangosteen (*Garcinia mangostana*) is currently becoming a popular tropical fruit, due to its high nutritional content of PTNs (especially phenolics and flavonoids) and antioxidant properties [6,7]. Furthermore, the lemongrass (*Cymbopogon citratus*) is increasing in popularity as a dietary supplement and that can be utilized in ruminant production as a rumen enhancer due to its content of PTNs and essential oils [8]. According to reports, the plant shows antibacterial, antioxidant, and the activities of anti-hyper ammonia-producing bacteria in the rumen [9]. The supplementation of lemongrass to the diet increased nutritional digestibility, while promoting beneficial ruminal fermentation [10], and reducing ruminal methane production [11]. Importantly, in this study, the novel technology was used as microencapsulation. This approach is frequently employed to preserve the functional and biological properties of PTN compounds and regulate their release [12].

Microencapsulation technique is a recent innovation that is frequently applied in the field of ruminant nutrition currently [13]. This method is utilized to enhance the stability, solubility, and bioavailability of PTNs. It is particularly useful for PTNs that are sensitive to environmental conditions, as it enables their enrichment in feed products [14]. One of microencapsulation methods, spray-drying is a widely used and cost-effective process that has several advantages, including high production rate, product quality, and encapsulation efficiency [15]. Nevertheless, the effects of microencapsulated lemongrass and mangosteen peel (M-LEMANGOS) combination as feed supplements on rumen fermentation and methane production are not widely investigated.

Therefore, the present study aimed to evaluate nutrient degradability, rumen fermentation, microbial diversity, and the production of methane *in vitro* receiving diets supplemented with M-LEMANGOS and crude protein (CP) levels.

## MATERIALS AND METHODS

### Ethics statement

The animals were subjected to review and permission by the Intuitional Animal and Use Committee of Khon Kaen University (protocol no. IACUC-KKU-110/66) and the Institute of Animals for Scientific Purpose Development (IAD), Thailand (approval number U1-06878-2560).

### Microencapsulated lemongrass and mangosteen peel preparation

**Figure 1:**
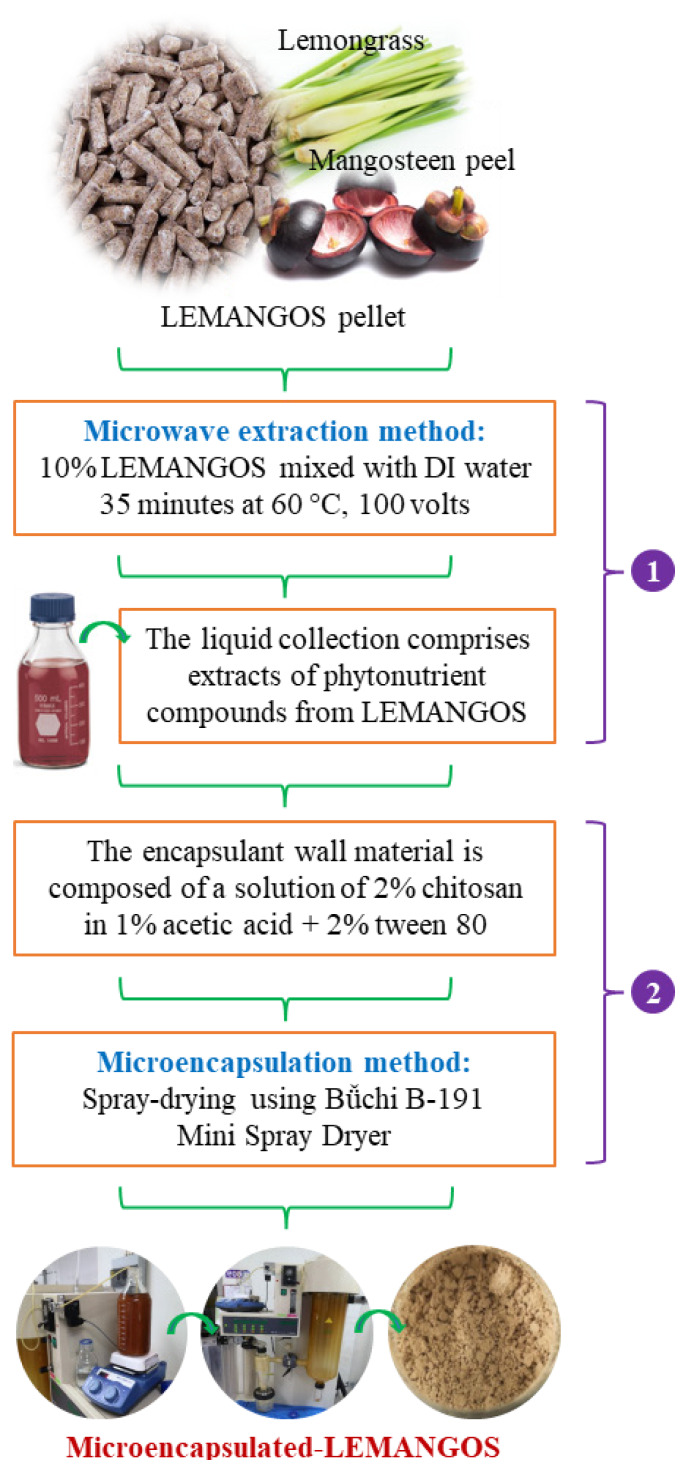
Diagram illustrating each step involved in the processing of microencapsulated-LEMANGOS formulation (1) procedure for the microwave extraction of phytonutrient compounds; (2) the technical processes for microencapsulation formulation. LEMANGOS, lemongrass and mangosteen peel.

Fresh lemongrass and mangosteen peel were sun-dried (2 to 3 days) then samples were prepared by grinding to a 1-mm sieve length (Cyclotech Mill; Tecator, Hoganas, Sweden). The sample and water were combined, microwaved for 35 minutes at 60°C, 100 volts, and the only liquids were collected. The wall-material components of chitosan at a concentration of 2% (w/v) were dissolved in a solution containing 1% (v/v) acetic acid and a surfactant (tween 80) at a concentration of 2% (v/v). The mixture was then stirred at a temperature of 65°C until it became consistent, and then was accomplished by combining wall material with PTN extract liquid in a 1:1 ratio (v/v) and stirring continuously at ambient temperature throughout the duration of the night. They were spray-dried with Bǚchi B-191 Mini Spray Dryer to form M-LEMANGOS according to the modified protocol of Phupaboon et al [16], the dried powders were kept at −20°C, as shown in Figure 1. Furthermore, the surface morphology of M-LEMANGOS was examined using a Field-emission scanning electron microscope (Mira; Tescan Co., Brno, Czech Republic).

### Feed chemical analyses

As presented in Table 1, following the procedures of AOAC [17], M-LEMANGOS, concentrate, and roughage were evaluated for dry matter (DM; no. 967.03), ash (no. 942.05), and CP (no. 984.13). The fiber fractions of the samples were analyzed using Ankom A200i Fibre Analyser (Ankom Technology Co., New York, NY, USA); following to van Soest et al [18]. Importantly, M-LEMANGOS was analyzed for i: PTNs namely total flavonoid compound (TFC) and total phenolic compound (TPC), ii: antioxidant capacities containing ferric reducing antioxidant power capacity, 2, 2′-azino-bis (3-ethylbenzothiazoline-6-sulfonic acid) (ABTS), and 2, 2-diphenyl-1-picrylhydrazyl (DPPH), these additional data have been reported in Phupaboon et al [16].

### Experimental design and treatments

The dietary treatments were allocated using a completely randomized design (CRD) in a 2×4 factorial arrangement, totally 8 treatments. Factor a was assessed as a %CP in the concentrate diet: 16% and 18%. The level of M-LEMANGOS is presented by factor B, which includes 0%, 2%, 4%, and 6% of total DM substrate, respectively.

### *In vitro* incubation

The protocols indicated by Menke and Steingass [19] were used for the *in vitro* analysis, artificial saliva preparation, and rumen fluid. In 50-mL bottles, the roughage to concentrate ratio was set at 60:40, with a weight of 0.5 g, then the M-LEMANGOS samples were weighed at the various levels of total substrate. The treatment bottle contained a volume of 40-mL of artificial saliva, along with the bottles containing rumen sample at the ratio of 2:1 (mL/mL). The medium solution (2,000-mL) is prepared by combining specific volumes of several solutions. These volumes are as follows: 0.24-mL of micro-mineral solution, 2.44-mL of resazurine, 99.0-mL of reduction solution, 480.0-mL of macro-mineral solution, 480.0-mL of buffer solution, and 950.0-mL of distilled water, respectively. Rumen fluid samples (four Thai-native beef cattle were used as a rumen donor, average body weight 400±20 kg) were obtained by introducing a tube, connected to a vacuum pump, through the oral cavity and into the middle part of the rumen. Subsequently, the samples were taken in a flask. The filtered samples were placed into a thermally insulated bottle at a temperature of 39°C, following the process of passing them through four layers of folded cheesecloth. Under constant CO_2_ flushing, all experimental bottles with aluminum lids were sealed with synthetic rubber stoppers, which were then incubated at 39°C.

### Gas determination

Gas production (24 bottles; 3 bottles/treatment×8 treatments) was recorded at 0, 1, 2, 4, 6, 8, 12, 24, 48, 72, and 96 h of incubation by using a 20-cc glass precision syringe to measure gas production using Fitcuve (software version 6, International Feed Resources Unit; MLURI, Aberdeen, UK) [20].

The bottles (32 bottles; 2 bottles/treatment×8 treatments×2 sampling times) were opened at 12 and 24 h and used for an immediately pH measurement (HI 8424 microcomputer; HANNA Instruments, Singapore). The rumen fluid samples were filtered through instances cheesecloth and centrifuged at 16,000×g for 15 minutes, the volatile fatty acid (VFA) and NH_3_-N will then be analyzed. VFA were used Gas chromatograph (GC; HP6890; Hewlett-Packard Co., Ltd., New York, NY, USA); using i: a front injector with injection volume of 1-μL and a syringe size of 10-μL, ii: an inlet with a pressure of 20.5 psi, a total flow rate of 12-mL/minutes, a heater set at a temperature of 200°C, iii: an oven with a temperature set at 120°C, and iv: an air flow rate of 400-mL/min, detector with a heater set at 200°C. The column used in the experiment was filled with a molecular sieve described as 13X, which had a mesh size of 30/60 (Alltech Associates Inc., Deerfeld, IL, USA), as more details provided by So et al [21]. NH_3_-N value was used a Spectrophotometer (UV/VIS; PG Instruments Ltd., London, UK). Nutrient degradability was set at 12 and 24 h (64 bottles; 2 bottles/treatment×8 treatments×2 sampling times×2 parameters). After passing the contents through pre-weighed Gooch crucibles with a porosity of 40-mm, the remaining DM was measured. The percent decrease in weight was calculated and reported as the *in vitro* dry matter degradability (IVDMD). The residues obtained from the incubation process were used an Ankom-bag (ANKOM 200; ANKOM Technology, Macedon, NY, USA). Then, feed sample that had been dried and the remaining residue were subjected to ash at 550°C in order to assess the IVOMD. Furthermore, methane production (32 bottles; 2 bottles/treatment×8 treatments×2 sampling times) was used GC machine (mL/0.5 g DM) (GC-2014; Model GC-17A System; Shimadzu Co., Ltd., Kyoto, Japan), fitted with a thermal conductivity detector (with a heater set at 140°C, injection temperature at 130°C) and a 2-m stainless steel column filled with Shin carbon (size 3-m×3-mm, column temperature at 120°C).

### Real-time polymerase chain reaction

From an *in vitro* investigation, approximately 1-mL of rumen fluid was collected for the extraction of total genomic DNA (gDNA) using the QIAamp Fast DNA Stool Mini kit method (Qiagen, Hilden, Germany). Employing a Nanodrop spectrophotometer (Thermo Scientific, Waltham, MA, USA), the absorbance at OD260/280 = 1.8 to 2.0 was used to determine the gDNA quality (the concentration at ≥50 ng/μL). Real-time polymerase chain reaction (PCR) was used to identify the microbial community using specific primers, namely *Ruminococcus albus* (Fs219f; GGTATGGGATGAGCTTGC, Fs654r; GCCTGCCCCTGAACTATC), *Ruminococcus flavefaciens* (Rf154f; TCTGGAAAC GGATGGTA, Rf425r; CCTTTAAGACAGGAGTTTACAA), *Fibrobactor succinnogenes* (Fs219f; GGTATGGGATGAGCTTGC, Fs654r; GCCTGCCCCTGAACTATC) [22], *Butyrivibrio proteoclasticus* (Bpf; CGWAGGGAAGCTGTTAAGT, Bpr; TACCGTCGTCCACTCCTT) [23], *Butyribrivio fibrisolvens* (Bff; CGCATG ATGCAGTGTGAAAAGCTC, Bfr; CCTCCCGACACCTATTATTCATCG) [24], *Megasphaera elsdenii* (Mef; GACCGAAACTGCGATGCTAGA, Mer; TCCAGAAA GCCGCTTTCGCCACT) [25], and *Methanobacteriales* (Mbt857f; GGG CTTGCTTTGGAAACTGTT, Mbt1196r; CCCACCGATGTTCCTCCTAA) [26], The amplification and detection of the real-time PCR were done by Luna Universal quantitative PCR Master Mix, additional details on the procedures were revealed in Koike and Kobayashi [22].

### Statistical analysis

A 2×4 Factorial arrangement in a CRD was used to investigate all of the experimental data using the PROC general linear model of Statistical analysis system (SAS; software version 9.4; SAS Institute, Cary, NC, USA) [27], as follows:


$${\text{Y}}_{\text{ij}}=\mu +\alpha_{\text{i}}+\beta_{\text{j}}+\alpha \beta_{\text{ij}}+{ɛ}_{\text{ij}}$$

where Y_ij_ = observation, μ = overall mean, α_i_ = level of %CP effect (i = 16% and 18% CP in concentrate), β_j_ = level of M-LEMANGOS effect (j = M-LEMANGOS at 0%, 2%, 4%, and 6% of total substrate), αβ_ij_ = effect of %CP×M-LEMANGOS, and ɛ_ij_ = error.

The difference between the means of the treatments at p<0.05 was calculated using Tukey’s multiple comparison test.

## RESULTS

### Morphological characterization and chemical composition of microencapsulated lemongrass and mangosteen peel

**Figure 2:**
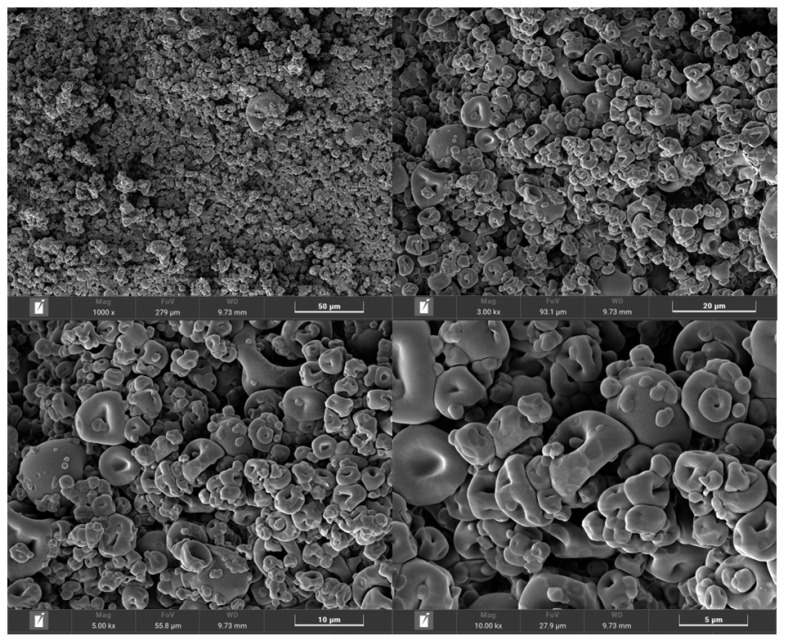
Morphological characterization of microencapsulated-LEMANGOS. LEMANGOS, lemongrass and mangosteen peel.

The morphology of microcapsules generated through the process of spray-drying. They offer various geometric shapes such as circles, squares, and surfaces that can be either smooth or rough. The microcapsules exhibit a binary structure, consisting of two concentric layers similar to a donut. The particle size ranges from 2 to 20-μm in diameter, as shown in Figure 2. Furthermore, the chemical composition of M-LEMANGOS were 88.7%, 18.6%, 43.1%, and 3.2% DM basis for organic matter, CP, acid-detergent fibe, and neutral-detergent fiber, respectively. Importantly, that product was consisted of PTNs especially TPCs (257.3-g GAE/kg DM) and TFCs (219.7-mg QUE/g DM). In part of antioxidant capacity, they contained 70.6% ABTS, 84.2% DPPH, and 20.6-g TROE/g DM). The results of M-LEMANGOS are presented in Table 1.

### Gas production kinetics and nutrient degradability

**Figure 3:**
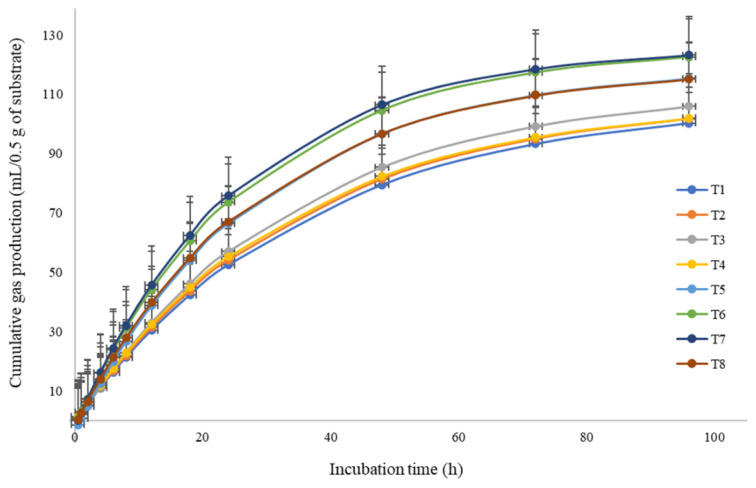
Cumulative gas production curves of the treatment diets in an *in vitro* study. Experimental treatments: T1, 16% crude protein (CP)+0% M-LEMANGOS; T2, 16% CP+2% M-LEMANGOS; T3, 16% CP+4% M-LEMANGOS; T4, 16% CP+6% M-LEMANGOS; T5, 18% CP+0% M-LEMANGOS; T6, 18% CP+2% M-LEMANGOS; T7, 18% CP+4% M-LEMANGOS; T8, 18% CP+6% M-LEMANGOS, respectively. M-LEMANGOS, microencapsulated phytonutrients from lemongrass and mangosteen peel. Error bars as mean±standard deviation.

The cumulative gas production curve is presented in Figure 3. The cumulative gas, gas *a*+*b*, gas *a*, gas *b*, and gas *c* were affected (p<0.01) by %CP added. M-LEMANGOS significantly increased (p<0.05) the gas *c* especially when supplemented at 4% to 6% of total substrate and the interaction between %CP and M-LEMANGOS was highly significant (p<0.01). Moreover, IVDMD and IVOMD both 12 and 24 h, and mean were enhanced (p<0.05) with M-LEMANGOS supplementation, while, there were similar with %CP (p>0.05; Table 2).

### Rumen fermentation characteristics

Regarding the VFA pattern in molar proportion, M-LEMANGOS addition significantly increased (p<0.05) propionate production at 12, 24 h, and mean (27.2, 28.6, and 27.9 mol/100 mL, respectively) in an *in vitro* study, with a higher value for M-LEMANGOS (4% of total substrate) than control group. The concentrations of acetate (12, 24 h, and mean) were significantly lower (p<0.05), while butyrate was not affected (p>0.05) when supplemented with M-LEMANGOS. The total VFA and acetate to propionate ratio both 12 and 24 h were significantly enhanced (p<0.01) at 4% of total substrate of M-LEMANGOS supplementation (Table 3).

As shown in Table 4, the various percents of CP in basal diet were affected by ruminal pH and NH_3_-N value, while M-LEMANGOS addition had no significant effect (p>0.05). The pH value is the range of 6.83 to 6.93. The concentration of NH_3_-N significantly decreased (p<0.01) with increasing levels of M-LEMANGOS, particularly at 4% to 6% total substrate. Moreover, CH_4_ production linearly decreased (p<0.01) with M-LEMANGOS supplementation, and there was lowest reduced with 4% to 6% M-LEMANGOS.

### Microbial population

The microbial population of *Ruminococcus albus, Ruminococcus flavefaciens, Fibrobacter succinogenes, Megasphaera elsdenii, Butyrivibrio fibrisolvens, Butyrivibrio proteoclasticus*, and *Methanobacteriales* showed a range of 8.7 to 9.8, 7.4 to 10.1, 8.3 to 11.9, 8.6 to 9.9, 7.9 to 9.1, 9.7 to 10.4, and 8.6 to 9.4 log copies of gene number/mL, respectively. The population of F*ibrobacter succinogenes, Megasphaera elsdenii*, and *Methanobacteriales* were significantly difference (p<0.05) by levels of CP in diets. *Fibrobacter succinogenes* and *Ruminococcus flavefaciens* population at 24 h were increased (p<0.05) with the interaction between %CP and M-LEMANGOS. Importantly, *Methanobacteriales* both 12 and 24 h were highly significant declined (p<0.05) with the interaction between %CP and M-LEMANGOS. No differences (p>0.05) were detected for *Ruminococcus albus, Butyrivibrio fibrisolvens*, and *Butyrivibrio proteoclasticus* when combining with %CP and M-LEMANGOS, as presented in Table 5.

## DISCUSSION

### Gas production kinetics and nutrient degradability

In the current experiment, gas production kinetics particularly gas c and cumulative gas were enhanced by the M-LEMANGOS addition. The potential reason for this phenomenon could be attributed by the presence of nutrients in M-LEMANGOS, which may facilitate the enhancement of gas c and cumulative gas production. M-LEMANGOS acquired energy through the utilization of cassava starch and molasses, while CP was attributed from lemongrass and mangosteen peel. Additionally, the formulation provided essential vitamins and minerals, thereby ensuring an adequate nutrient supply to support microbial activity during the incubation process. The utilization of pellet form, which involves the synchronization of fermentation with degradable carbohydrates and nitrogen sources, resulted in a higher level of microbial activity in the rumen as compared to diets that was not incorporated in the pellets [28]. Hence, in order to enhance microbial synthesis, it becomes imperative to initially focus on manipulating carbohydrate and nitrogen fermentation in the rumen to achieve the most uniform rumen carbohydrate supply pattern feasible within a particular dietary regimen [29].

The IVDMD and IVOMD at 12 and 24 h were affected by increasing level of M-LEMANGOS. Specifically, the inclusion of M-LEMANGOS at 4% of the total substrate resulted in significantly increased nutrient degradability values. The potential impact of PTN content in M-LEMANGOS on enhancing microbial activity in the rumen may be elucidated. PTNs have the potential to function as probiotics by enhancing the proliferation of gut microorganisms, so facilitating the digestion of feed [30]. According to Norrapoke et al [31], who showed that mangosteen peel supplementation could increase gas production kinetics and IVDMD, respectively.

### Rumen fermentation characteristics

The VFAs are the end-products of rumen fermentation, and alterations in their composition due to the inclusion of feed additives can indicate the effects of the supplementation on the rumen environment [32]. The supplementation of M-LEMANGOS resulted in an obvious shift in the VFA profile when compared to the control group. They induced a change in ruminal fermentation, resulting in an increase in the percentage of propionate production. Wettstein et al [33] stated that reduction in ruminal methanogenesis can lead to a shift in rumen fermentation from acetate to propionate, after that this shift occurs due to the preferential activation of the pathway of propionate synthesis rather than the acetate synthesis pathway. In the rumen, the process of CH_4_ generation is generally regarded as a symbiotic relationship between microorganisms that produce hydrogen (H_2_) and methanogens that consume H_2_, consequently, the management of the H_2_ sink plays a significant role in reducing CH_4_ emissions [34]. One effective option for mitigating the major metabolic H_2_ emissions is to increase propionate synthesis. This approach serves as a sink for H_2_ and CH_4_, hence reducing the availability of H_2_ for methanogens [35]. In the current investigation, it was observed that the addition of M-LEMANGOS significantly decreased CH_4_ production as compared to the control group at 12 and 24 h of incubation time. The experiment conducted by Jafari et al [36] indicated that the peels of various tropical fruits containing phenolics, including dokong, mangosteen, papaya, pineapple, and rambutan, exhibited a reduction in CH_4_ production *in vitro*. Kholif et al [30] stated that the supplementation of lemongrass to the diet resulted in enhancements in the generation of ruminal propionate and total VFAs. Some studies have documented these PTNs (phenolics and flavonoids) inhibit methanogens directly and/or suppress the microbial metabolic pathways that are involved in methanogenesis, which is accompanied with notable improvements in specific fermentation parameters. Hence, it is plausible that substances derived from numerous tropical plants possess the capability to directly inhibit the growth and function of methanogens, either independently or in combination [37].

The establishment of an optimal rumen environment is essential for the attainment of efficient performance in ruminants. The rumen pH has a significant role in determining the stability of the rumen environment, as it can lead to changes in microbial populations and is necessary for the optimal growth of microorganisms [38]. The addition of M-LEMANGOS remained in a consistent ruminal pH level ranging from 6.83 to 6.93. Wanapat [39] reported that microbial activity and growth are most beneficial within the pH range of 6.5 to 7.0. The potential for enhancing rumen efficiency by the administration of PTN lies in its ability to maintain a higher pH, increase NH_3_-N content, and stimulate microbial protein synthesis [40]. Furthermore, the combination of M-LEMANGOS and %CP in concentrate lowered the NH_3_-N concentration. The observed phenomenon can be attributed by the presence of the PTN in M-LEMANGOS, which has the ability to decelerate the processes of ruminal proteolysis, peptidolysis, and deamination [41]. PTNs present in M-LEMANGOS have the ability to protect proteins against degradation, hence enhancing nitrogen utilization efficiency in ruminants. This is achieved through two mechanisms: firstly, by uplifting the quantity of by-pass protein, and secondly, by decreasing fiber degradation in the rumen through the inhibition of microbial attachment to feed particles. These findings agreed with Calabrò et al [42] and Guglielmelli et al [43] revealed that the optimal PTN level would contribute to the protection of proteins from rumen digestion, thereby leading to an increase in bypass protein. Wanapat et al [44] also demonstrated that MARABAC pellet, consisting of a composition of mangosteen peel, rambutan peel, and banana flower powder, has enormous potential as a dietary rumen enhancer. It offers a viable alternative to the use of chemicals and antibiotics traditionally employed to improve rumen fermentation, particularly in terms of pH maintenance and reduction of NH_3_-N concentration.

### Microbial population

*Fibrobacter succinogenes, Ruminococcus flavefaciens*, and *Megasphaera elsdenii* population were increased with the interaction between %CP in concentrate and M-LEMANGOS supplementation. This could be due to M-LEMANGOS containing PTN and may affect to improve microbial population. In relation to microbial populations, PTNs exhibit diverse biological characteristics that can potentially induce bacterial proliferation or modify ruminal microorganisms, consequently impacting feed digestion in the rumen [45]. The supplementation of PTNs, which included phenolic acids and flavonoids, was observed to result in an increase in both the overall abundance of bacteria and the specific populations of certain species/genera of bacteria, namely *F. succinogenes* and *B. fibrisolvens* [46]. Moreover, *Methanobacteriales* population was decreased and they play an important role in the effectiveness of rumen methanogenesis. The suppression of rumen methanogenesis is typically accompanied by a concurrent elevation in propionate concentration, which was also observed in the present experiment. Ma et al [47] reported that flavonoids addition from tropical plat decrease ruminal populations of methanogens. Phesatcha et al [48], in addition demonstrated that the inclusion of mangosteen peel in the diet resulted in a reduction in the population of methanogens. Under currently investigation, Matra et al [49] stated that microencapsulated-PTNs from *Mitragyna* plant could improve cellulolytic bacteria especially increase the population of *B. fibrisolvens*, and decrease methanogens.

## CONCLUSION

The inclusion of M-LEMANGOS at 4% of total substrate and 18% CP of concentrate resulted in enhanced degradability of nutrients and improved rumen enteric fermentation by increasing propionate, narrowing acetate to propionate ratio, nutrient degradability, and mitigating methane production. Thus, M-LEMANGOS has the potential to serve as a viable tropical plant-based PTN for the purpose of modulating rumen fermentation. An *in vivo* assessment should be undertaken to elucidate insightful data.

## Figures and Tables

**Table 1:** Ingredient and chemical composition of the basal diet

Items	Concentrate		Rice straw	M-LEMANGOS

	16% CP	18% CP		
Ingredients (% as fed)				
Cassava chip	40.0	40.2		
Rice bran meal	11.8	12.0		
Palm kernel meal	11.0	12.0		
Soybean meal	19.0	18.5		
Corn meal	12.0	10.3		
Molasses	3.0	3.0		
Urea	1.2	2.0		
Mineral premixed^1)^	1.0	1.0		
Salt	0.5	0.5		
Di-calcium	0.5	0.5		
Chemical composition (% dry matter)				
Dry matter (DM, %)	89.9	89.5	89.4	82.8
Organic matter (OM)	94.4	93.9	85.4	88.7
Crude protein (CP)	16.3	18.3	2.4	18.6
Neutral-detergent fiber (NDF)	20.8	22.9	78.9	43.1
Acid-detergent fiber (ADF)	11.7	13.0	52.6	3.2
Phytonutrient compound				
TPC (g GAE/kg DM)	-	-	-	2.6
TFC (g QUE/kg DM)	-	-	-	0.2
Antioxidant capacity				
ABTS (%)	-	-	-	70.6
DPPH (%)	-	-	-	84.2
FRAP (g TROE/g DM)	-	-	-	20.6

M-LEMANGOS, microencapsulated phytonutrients from lemongrass and mangosteen peel; CP, crude protein; TPC, total phenolic compounds; TFC, total flavonoid compounds; ABTS, 2’-azino-bis (3-ethylbenzothiazoline-6-sulfonic acid); DPPH, 2, 2-diphenyl-1-picrylhydrazyl; FRAP, ferric reducing antioxidant power.

1) Mineral premixed (contains per kg): vitamin A 10,000,000 IU; vitamin D 1,600,000 IU; vitamin E 70,000 IU; Fe 50 g; Mn 40 g; Zn 40 g; Cu 10 g; I 0.5 g; Se 0.1 g; Co 0.1 g.

**Table 2:** Gas production kinetics and nutrient degradability of the treatment diets in an *in vitro* study

Treatments	%CP in concentrate	M-LEMANGOS (% of total substrate)	Gas production kinetics^1)^				Cumulative gas at 96 h^2)^	IVDMD (% DM)		IVOMD (% DM)	
		
			*a*	*b*	*c*	*a+b*		12 h	24 h	12 h	24 h
1	16	0	−0.5^a^	108.1^e^	0.028^e^	107.6^e^	106.1^d^	56.7^c^	62.2^c^	64.9^d^	71.7^d^
2	16	2	−0.8^b^	109.3^e^	0.029^d^	108.5^d^	107.4^d^	58.1^b^	64.1^b^	66.3^b^	73.1^b^
3	16	4	−1.4^c^	113.7^d^	0.030^d^	112.3^c^	111.2^c^	59.3^b^	65.6^a^	67.1^a^	73.9^a^
4	16	6	−0.4^a^	108.4^e^	0.030^d^	108.0^d^	107.9^d^	53.3^d^	60.9^d^	65.6^c^	72.4^c^
5	18	0	−3.6^f^	123.2^b^	0.035^c^	119.6^b^	120.4^b^	56.9^c^	60.1^d^	66.1^b^	72.9^b^
6	18	2	−1.7^d^	127.9^a^	0.037^b^	126.2^a^	127.8^a^	59.0^b^	63.8^b^	66.6^b^	73.4^b^
7	18	4	−2.5^e^	128.7^a^	0.039^a^	126.2^a^	126.1^a^	61.2^a^	65.9^a^	67.4^a^	74.2^a^
8	18	6	−1.8^d^	121.1^c^	0.035^c^	119.3^b^	119.4^b^	51.9^e^	58.1^e^	66.5^b^	73.3^b^
SEM			0.32	1.72	0.01	1.95	1.78	1.12	1.15	0.87	0.93
Comparisons											
%CP			<0.01	<0.01	<0.01	<0.01	<0.01	0.35	0.14	0.18	0.06
M-LEMANGOS			0.09	0.72	0.04	0.47	0.72	0.03	0.04	0.01	<0.01
Interaction			0.24	0.15	<0.01	0.15	0.10	0.94	0.81	0.76	0.59

CP, crude protein; M-LEMANGOS, microencapsulated phytonutrients from lemongrass and mangosteen peel; IVDMD, *in vitro* dry matter degradability; DM, dry matter; IVOMD, *in vitro* organic matter degradability; SEM, standard error of mean.

1) Gas production kinetics, *a*, the gas production from the immediately soluble fraction (mL); *b*, the gas production from the insoluble fraction (mL); c, the gas production rate constant for the insoluble fraction (mL/h); *a*+*b*, the potential extent of gas production (mL).

2) Cumulative gases at 96 h (ml/0.2 g DM substrate).

a–f Means within the same column with different letters are significantly different at p<0.05.

**Table 3:** Volatile fatty acid profiles of the treatment diets in an *in vitro* study

Treatments	%CP in concentrate	M-LEMANGOS (% of total substrate)	Volatile fatty acids (mol/100 mL)						Acetate to propionate ratio		Total volatile fatty acids (mmol/L)	

			Acetate		Propionate		Butyrate					
				
			12 h	24 h	12 h	24 h	12 h	24 h	12 h	24 h	12 h	24 h
1	16	0	66.5^a^	65.9^a^	23.4^e^	24.8^e^	10.2	9.4	2.8^b^	2.7^b^	102.4^g^	105.2^g^
2	16	2	65.7^b^	65.1^b^	23.6^e^	25.0^e^	10.7	9.9	2.8^b^	2.6^c^	105.2^e^	108.0^e^
3	16	4	64.3^c^	63.7^d^	26.5^b^	27.9^b^	9.2	8.4	2.4^e^	2.3^f^	112.3^b^	115.1^b^
4	16	6	66.6^a^	66.0^a^	22.4^f^	23.8^f^	11.0	10.2	3.0^a^	2.8^a^	103.5^f^	106.3^f^
5	18	0	65.5^b^	64.9^b^	24.2^d^	25.6^d^	10.3	9.5	2.7^c^	2.5^d^	105.3^e^	108.2^e^
6	18	2	64.7^c^	64.1^c^	25.4^c^	26.8^c^	9.9	9.1	2.5^d^	2.4^e^	107.6^c^	110.5^c^
7	18	4	64.1^c^	63.5^d^	27.2^a^	28.6^a^	8.7	7.9	2.4^e^	2.2^g^	118.7^a^	121.6^a^
8	18	6	65.8^b^	65.2^b^	24.0^d^	25.4^d^	10.2	9.4	2.7^c^	2.6^c^	105.8^d^	108.7^d^
SEM			0.64	0.52	0.45	0.51	0.86	0.94	0.08	0.09	0.54	0.57
Comparisons												
%CP			0.74	<0.01	0.07	0.04	0.92	0.98	0.06	0.07	<0.01	<0.01
M-LEMANGOS			0.03	<0.01	<0.01	0.02	1.24	1.52	<0.01	<0.01	<0.01	<0.01
Interaction			0.56	0.67	0.95	0.33	0.53	0.79	0.93	1.14	0.24	0.32

CP, crude protein; M-LEMANGOS, microencapsulated phytonutrients from lemongrass and mangosteen peel; SEM, standard error of mean.

a–g Means within the same column with different letters are significantly different at p<0.05.

**Table 4:** Rumen fermentation characteristics of the treatment diets in an *in vitro* study

Treatments	%CP in concentrate	M-LEMANGOS (% of total substrate)	pH		Ammonia-nitrogen (mg/dL)		Methane production (mL/0.5 g DM)	
		
			12 h	24 h	12 h	24 h	12 h	24 h
1	16	0	6.91^c^	6.84^e^	17.8^c^	18.5^e^	4.5^a^	9.3^a^
2	16	2	6.86^e^	6.84^e^	17.8^c^	18.2^f^	3.9^b^	9.1^a^
3	16	4	6.86^e^	6.85^d^	16.0^e^	18.1^f^	2.7^c^	7.2^b^
4	16	6	6.87^d^	6.83^f^	15.9^e^	17.8^g^	2.4^f^	4.4^f^
5	18	0	6.91^c^	6.85^d^	18.9^a^	21.6^a^	2.6^d^	7.4^b^
6	18	2	6.92^b^	6.86^c^	18.6^b^	20.7^b^	2.5^e^	6.9^c^
7	18	4	6.93^a^	6.90^a^	17.8^c^	20.5^c^	2.4^f^	5.7^d^
8	18	6	6.91^c^	6.87^b^	17.6^d^	20.0^d^	2.4^f^	5.0^e^
SEM			0.01	0.01	0.30	0.30	0.28	0.47
Comparisons								
%CP			<0.01	<0.01	0.04	<0.01	0.63	0.26
M-LEMANGOS			0.17	0.06	0.91	0.95	<0.01	<0.01
Interaction			0.06	0.11	0.20	0.56	0.05	0.65

CP, crude protein; M-LEMANGOS, microencapsulated phytonutrients from lemongrass and mangosteen peel; DM, dry matter; SEM, standard error of mean.

a–g Means within the same column with different letters are significantly different at p<0.05.

**Table 5:** Microbial population of the treatment diets in an *in vitro* study

Treatments	%CP in concentrate	M-LEMANGOS (% of total substrate)	Microbial population (Log copies of gene number/mL)													

			FS		RA		RF		ME		BF		BP		MET	
						
			12 h	24 h	12 h	24 h	12 h	24 h	12 h	24 h	12 h	24 h	12 h	24 h	12 h	24 h
1	16	0	8.7	8.3^g^	9.2	9.4	8.8	7.9^e^	9.0^c^	9.2	8.6	6.5	10.0	10.2	9.3^a^	9.3^b^
2	16	2	8.7	8.6^f^	9.5	9.5	8.9	8.9^c^	9.1^b^	9.3	9.1	7.9	9.9	10.1	9.3^a^	9.3^b^
3	16	4	8.8	8.8^e^	9.7	9.5	9.2	9.0^c^	9.1^b^	9.3	8.2	8.5	9.9	9.8	9.2^b^	9.1^c^
4	16	6	8.5	8.6^f^	9.3	8.8	8.3	7.4^g^	9.4^a^	9.2	9.1	8.1	9.9	9.9	9.2^b^	9.0^d^
5	18	0	8.5	9.3^d^	9.3	9.2	8.8	8.7^d^	8.6^g^	9.1	9.0	8.5	10.0	10.1	9.3^a^	9.4^a^
6	18	2	8.7	9.8^c^	9.4	9.5	9.0	9.7^b^	8.7^f^	9.2	8.9	8.5	10.1	9.7	9.2^b^	8.8^e^
7	18	4	8.8	11.9^a^	9.7	9.8	9.5	10.1^a^	8.9^d^	9.4	8.8	8.3	10.1	10.4	9.1^c^	8.7^f^
8	18	6	8.7	11.0^b^	8.7	9.3	9.4	7.6^f^	9.0^c^	9.9	8.4	8.4	9.8	10.1	8.9^d^	8.6^g^
SEM			0.05	0.20	0.08	0.10	0.10	0.23	0.07	0.08	0.10	0.30	0.03	0.07	0.02	0.05
Comparisons																
%CP			0.90	<0.01	0.36	0.50	0.08	0.15	0.03	0.22	0.85	0.27	0.57	0.52	0.05	0.02
M-LEMANGOS			0.65	0.09	0.07	0.20	0.70	0.70	0.35	0.38	0.43	0.72	0.33	0.53	0.04	0.08
Interaction			0.32	0.04	0.70	0.80	0.09	0.04	080	0.30	0.20	0.60	0.33	0.10	0.02	0.03

CP, crude protein; M-LEMANGOS, microencapsulated phytonutrients from lemongrass and mangosteen peel; FS, *Fibrobacter succinogene*s; RA, *Ruminococcus albus*; RF, *Ruminococcus flavefaciens*; ME, *Megasphaera elsdenii*; BF, *Butyrivibrio fibrisolvens*; BP, *Butyrivibrio proteoclasticus*; MET, Methanobacteriales; SEM, standard error of mean.

a–g Means within the same column with different letters are significantly different at p<0.05.
